# Fibrinolytic-deficiencies predispose hosts to septicemia from a catheter-associated UTI

**DOI:** 10.21203/rs.3.rs-3263501/v1

**Published:** 2023-09-15

**Authors:** Jonathan J. Molina, Kurt N. Kohler, Christopher Gager, Marissa J. Andersen, Ellsa Wongso, Elizabeth R. Lucas, Andrew Paik, Wei Xu, Deborah L. Donahue, Karla Bergeron, Aleksandra Klim, Michael G. Caparon, Scott J. Hultgren, Alana Desai, Victoria A. Ploplis, Matthew J. Flick, Francis J. Castellino, Ana L. Flores-Mireles

**Affiliations:** 1 Integrated Biomedical Sciences, University of Notre Dame, IN 46556, USA; 2 Department of Biological Sciences, University of Notre Dame, Notre Dame, IN 46556, USA.; 3 W. M. Keck Center for Transgene Research, University of Notre Dame, Notre Dame, IN 46556, USA.; 4 Department of Chemistry and Biochemistry, University of Notre Dame, Notre Dame, IN 46556, USA.; 5 Department of Molecular Microbiology, Washington University School of Medicine, MO 63110, USA,; 6 Center for Women’s Infectious Disease Research, Washington University School of Medicine, MO 63110, USA; 7 Department of Surgery, Washington University School of Medicine, MO 63110, USA.; 8 Department of Urology, University of Washington Medical Center, WA 98133-9733, USA.; 9 Department of Pathology and Laboratory Medicine, University of North Carolina, Chapel Hill, NC 27599, USA; 10 UNC Blood Research Center, University of North Carolina, Chapel Hill, NC 27599, USA; 11 Equal-contribution; 12 Lead contact

**Keywords:** CAUTI, *E. faecalis*, *E. coli*, *C. albicans*, inflammation, polymicrobial, fibrin(ogen), fibrinolysis, dissemination, bacterial proteases

## Abstract

Catheter-associated urinary tract infections (CAUTIs) are amongst the most common nosocomial infections worldwide and are difficult to treat due to multi-drug resistance development among the CAUTI-related pathogens. Importantly, CAUTI often leads to secondary bloodstream infections and death. A major challenge is to predict when patients will develop CAUTIs and which populations are at-risk for bloodstream infections. Catheter-induced inflammation promotes fibrinogen (Fg) and fibrin accumulation in the bladder which are exploited as a biofilm formation platform by CAUTI pathogens. Using our established mouse model of CAUTI, we identified that host populations exhibiting either genetic or acquired fibrinolytic-deficiencies, inducing fibrin deposition in the catheterized bladder, are predisposed to severe CAUTI and septicemia by diverse uropathogens in mono- and poly-microbial infections. Furthermore, we found that *E. faecalis*, a prevalent CAUTI pathogen, uses the secreted protease, SprE, to induce fibrin accumulation and create a niche ideal for growth, biofilm formation, and persistence during CAUTI.

## INTRODUCTION

Urinary catheterization is a common procedure to drain urine from patients’ bladders due to chronic conditions or while in healthcare facilities, intensive care units, during surgical procedures and recovery^1–3^. Despite its benefits, catheter placement increases the risk of developing a catheter-associated urinary tract infection (CAUTI)^4–7^. CAUTIs are one of the most common nosocomial infections and often lead to septicemia with a 30% mortality^3,8^. In fact, ~25% of sepsis cases come from complicated UTI, including CAUTIs^9^. Current CAUTI management focuses on catheter removal, replacement, and an antibiotic regiment^6^. However, an increasing prevalence of antibiotic resistant CAUTI pathogens imposes challenges to management and treatment^6,10,11^ and are classified as serious threats by the CDC^12^ and WHO^13^.

Currently, there is no consensus on best practices for CAUTI treatment. Patients are treated with the same protocols as non-catheter associated (uncomplicated) urinary tract infections (uUTI). However, CAUTIs exhibit unique clinical manifestations, causative organisms, and pathologic mechanisms, making these infections distinct from uUTI^6^. For example, uUTIs are more prevalent in women than men (4:1 ratio) while in CAUTI there is no gender bias^6^. Also, *E. coli* accounts for >95% of uUTI, whereas CAUTI-pathogens are more diverse, including gram-negative, gram-positive, and fungal pathogens^6^.

In both humans and mice, urinary catheterization provokes local tissue damage to the bladder, activating coagulation, and converting the blood coagulation protein, fibrinogen (Fg), into fibrin. Fibrin clots and extravascular fibrin deposits promote blood clotting and wound healing^14^. Then, in fibrinolysis, plasminogen (Pg) is activated into serine protease plasmin to degrade fibrin clots and restores tissue homeostasis^14,15^. However, constant catheter-induced bladder inflammation induces Fg/fibrin accumulation on the catheter with increasing catheterization time in human and mice^16,17^. Fg/fibrin deposition compromises the urothelium, thereby introducing a platform for biofilm formation by CAUTI pathogens^16–23^.

Here, we identified that host populations with genetic or acquired fibrinolytic-deficiencies are susceptible to severe and persistent CAUTI and systemic dissemination by diverse groups of prevalent uropathogens. Moreover, we found that *E. faecalis*, a prevalent CAUTI pathogen, secretes a protease, SprE, to induce fibrin accumulation and create a niche ideal for growth, biofilm formation and persistence during CAUTI. Furthermore, antifibrinolytic agents usage in catheterized mice results in Fg/fibrin accumulation in the bladders, resulting in persistent CAUTI and systemic dissemination in mono- and poly-microbial infection by *E. faecalis*, *E. coli*, and *C. albicans*. Identification of patient populations with higher susceptibility to CAUTI and its sequelae will allow clinicians to improve patient outcomes by implementing efficient patient monitoring to mitigate infection incidences, morbidities, and mortality.

## RESULTS

### Prolonged urinary catheterization promotes enterococcal burden and persistence in the bladder and systemic dissemination.

**Figure 1.**
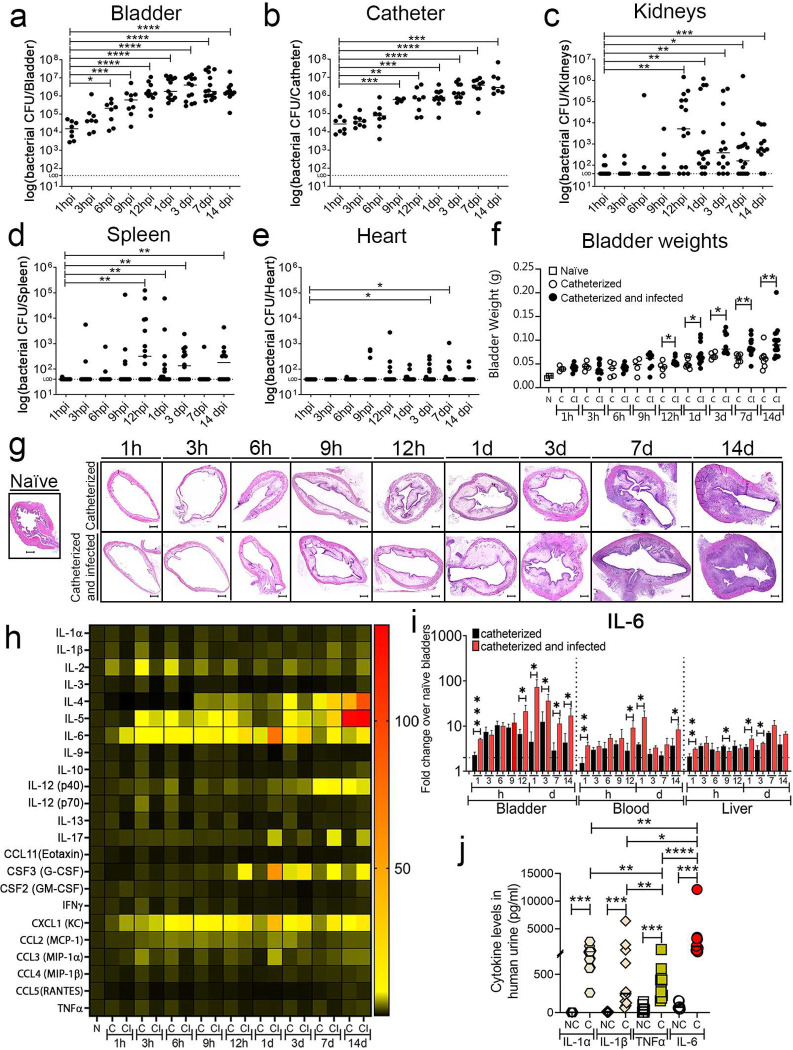
*E. faecalis* infection and inflammation progression during urinary catheterization. **(a-e)** *E. faecalis* establishes persistent colonization overtime. Mice were catheterized and infected with 2×10^7^ CFU of *E. faecalis* OG1RF. Enterococcal colonization of organs and catheters were assessed by quantifying bacterial burden. **(f)** Bladder weights of naïve (non-implanted control) mice and implanted mice in the presence or absence of the *E. faecalis* at the indicated times. **(g)** Bladder sections were stained with H&E to compare inflammation from catheterization in the presence or absence of the pathogen overtime (scale bars: 500 μm). **(h)** Heatmap represents catheterized bladder cytokines fold changes over naïve bladder in the presence or absence of the *E. faecalis* OG1RF infection at the indicated times. (**i**) IL-6 analysis levels in the catheterized bladder (with or without infection), bloodstream, and liver. The horizontal broken line represents twofold change cytokine levels compared with naïve control mice over a 2-fold change. (**j**) Comparison of the IL-1α, IL-1β, IL-6, and TNFα levels in urine from patients catheterized for 24h and healthy donors (NC; non-catheterized). Values represent means ± SEM. Values represent median. The Mann-Whitney U test was used; *, P < 0.05 was considered statistically significant. **, P < 0.005; ***, P < 0.0005; ****, P<0.0001. The horizontal bar represents the median value. The horizontal broken line represents the limit of detection of viable bacteria. LOD; limit of detection. Infections were done in three independent experiments with n = 6 mice for each one, and data are shown as the log (bacterial CFU/organ or catheter). Animals that lost the catheter were not included in this work.

Clinical studies showed that CAUTI-burdened patients have an increased risk of septicemia and mortality^10,11,24–26^. To understand *E. faecalis* bladder and catheter colonization progression and systemic dissemination, we performed a temporal study examining acute infection (1, 3, 6, 9, 12 hr post-catheterization and infection (hpi) and 1 day post-infection (dpi)) and prolonged infection (3, 7, 14 dpi). Female C57BL/6Ncr mice were catheterized and infected with of *E. faecalis* (~2 × 10^7^ CFU) or mock-infected (PBS). At specified timepoints, mice were sacrificed to harvest the bladders, catheters, kidneys, spleens, and hearts. Bladders at 1 hpi have an initial colonization of ~10^4^ bacterial CFU that increased significantly overtime, showing maximum colonization at ~10^7^ CFU by 12 hpi in the bladder and persisting through 14 dpi (Fig. 1a). Similarly, catheter colonization significantly increased with catheterization time and persisted (Fig. 1b). Importantly, robust bladder and catheter colonization allowed *E. faecalis* to disseminate to the kidneys, spleen, and heart (Fig. 1c–e). Thus, our data recapitulates what is clinically observed.

### Progression of bladder inflammation correlates with microbial burden.

**Extended Data Figure 1.**
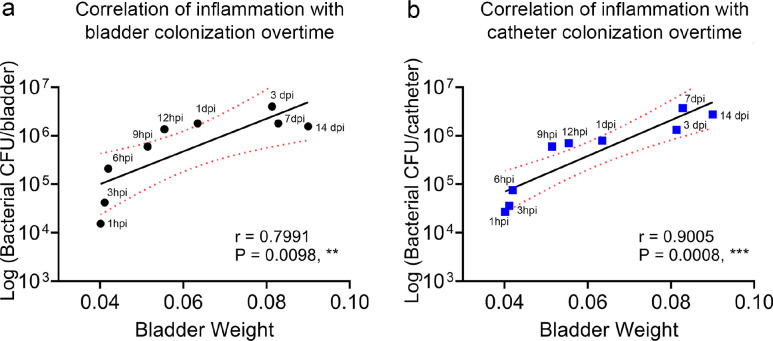
Catheter-induced inflammation correlates with pathogen colonization overtime. Pearson’s correlation statistical analysis was used to quantify the association between the catheter-induced bladder inflammation and pathogen colonization in the bladder (**a**) or catheter (**b**) overtime. For this test, median bladder weights values from catheterized and infected mice and the median values of the CFUs of bladders or catheters were used to perform this test. r, Pearson’s correlation coefficient. *, P < 0.05 was considered statistically significant. **, P < 0.005; ***, P < 0.0005.

Catheter-induced inflammation is a CAUTI hallmark^21,27,28^. To understand how urinary catheterization changes the bladder environment during acute and prolonged catheterization and in the presence or absence of infection; we examined mouse bladder edema by bladder weight and histological analysis. Bladders exhibited a progressive weight increase with the catheter dwell time (Fig. 1f). Bladder histological analysis corroborates the gradual edema progression associated with urinary catheterization (Fig. 1g). Furthermore, there is a significant positive correlation of bladder inflammation with bacterial burden in the bladder (r= 0.7991; P= 0.0098) or catheter (r= 0.9005; P= 0.0008) (Extended Data Fig. 1). Importantly, *E. faecalis* infection further increased edema after 12 hpi (Fig. 1f). This indicates that catheterization alone increases bladder edema overtime and is exacerbated by an *E. faecalis* infection.

### *E. faecalis* presence modulates levels of inflammatory cytokines in the catheterized bladder.

**Extended Data Figure 2.**
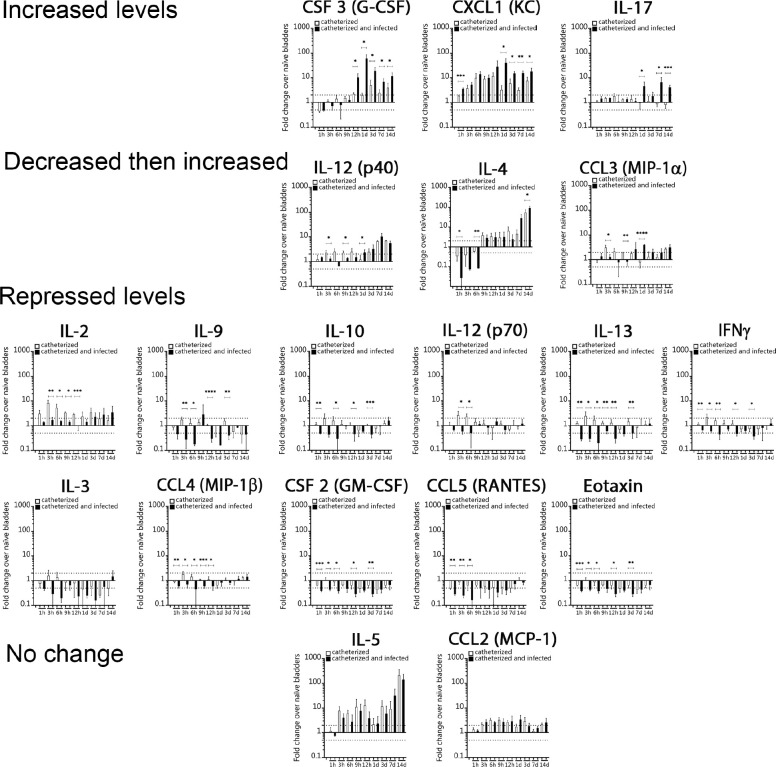
Inflammatory cytokines of the catheterized bladder. Analysis of individual cytokine levels in the catheterized bladder (with or without *E. faecalis* infection) that increased, decreased then decreased, were repressed, or had no change. The horizontal broken line represents 2-fold change cytokine levels compared with naïve control mice. Values represent means ± SEM. The Mann-Whitney U test was used; *, P < 0.05 was considered statistically significant. **, P < 0.005; ***, P < 0.0005; ****, P<0.0001. Animals that lost the catheter were not included in this work.

**Extended Data Figure 3.**
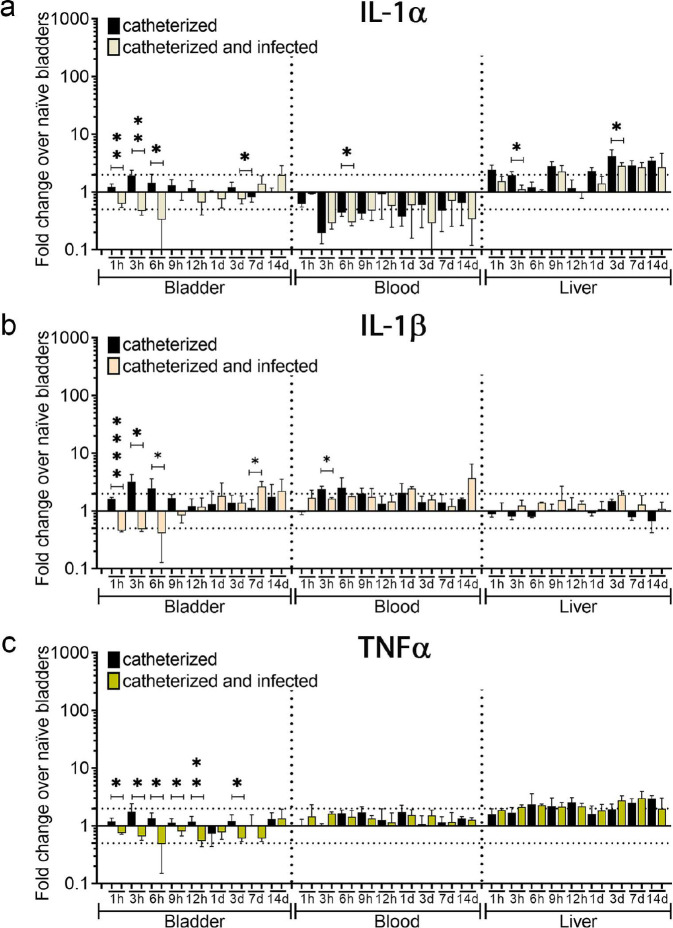
Comparative analysis of 1L-1α, IL-1β, and TNFα levels. 1L-1α (**a**), IL-1β (**b**), and TNFα (**c**) levels in the catheterized bladder (with or without infection), bloodstream, and liver. The horizontal broken line represents twofold change cytokine levels compared with naïve control mice over a 2-fold change. Values represent means ± SEM. Values represent median. The Mann-Whitney U test was used; *, P < 0.05 was considered statistically significant. **, P < 0.005; ****, P<0.0001.

Previous studies show that acute urinary catheterization induces many inflammatory cytokines in the bladder^27^, of which IL-1β, IL-6, 12(p40), IL-17, CSF 3, and CXCL1 are further induced during *E. faecalis* infection^28^. Since *E. faecalis* significantly exacerbates bladder inflammation during catheterization (Fig. 1f–g), we profiled cytokine level changes of *E. faecalis-* or mock-infected catheterized bladder, relative to naïve bladders. We found that *E. faecalis* infection altered cytokine expression patterns differently than in mock-infected bladders. IL-6, CSF 3, CXCL1, and IL-17 levels were significantly higher during infection (Fig. 1h,i, and Extended Data Fig. 2). Conversely, infection significantly reduced levels of IL-1α, IL-2, IL-9, IL-10, IL-12 (p70), IL-13, IFNγ, IL-3, CCL4 (MIP-1β), CSF 2 (GM-CSF), CCL5 (RANTES), and TNFα (Fig. 1h, Extended Data Fig. 2 and 3a,c). IL-1β, IL-12 (p40), IL-4, and CCL3 levels were modulated, showing significantly lower induction during early infection (1–9 hrs) followed by a significant increased after 12hrs (Fig. 1h, Extended Data Fig. 2 and 3b). IL-5 and CCL2 levels increased regardless of the infection (Fig. 1h and Extended Data Fig. 2). This indicates that there is a differential inflammatory cytokine profile on infected catheterized bladders.

During catheter-induced bladder inflammation in mice and humans^16,17^, Fg, a liver-produced proinflammatory protein, is recruited into the bladder and deposited on urinary catheters, serving as a platform for microbial CAUTI^16,18,21–24,29^. IL-1 (α and β), IL-6, and TNFα are known to stimulate Fg production by migrating from damaged tissues, through the bloodstream and signaling to Fg-producing hepatocytes in the liver^30,31^. To understand their contribution to CAUTI-associated inflammation, we evaluated levels of these proinflammatory mediators in the bladder. High levels of IL-6 during catheterization were observed, which significantly increased during infection (Fig. 1h,i). We also assessed the levels of all four cytokines in the bloodstream and livers. Expectedly, IL-6 levels were elevated in the bloodstream and liver (Fig. 1i and Extended Data Fig. 3). To understand the role of IL-1α, IL-1β, IL-6 and TNFα in human urinary catheterization, we collected urine from patients that had a urinary catheter for ~24 hrs and from healthy donors to compare and analyze their levels. We found that IL-1α, IL-1β, IL-6 and TNFα levels significantly increased in urine from catheterized patients when compared with urine from non-catheterized healthy donors (Fig. 1j). Furthermore, IL-6 was significantly elevated compared with IL-1α, IL-1β, and TNFα in the catheterized patients (Fig. 1j). Suggestively, IL-6 is the main cytokine that signals the liver Fg expression and release, resulting in its accumulation in the catheterized bladder.

### Fg deposition increases on urinary catheters during *E. faecalis* infection.

**Figure 2.**
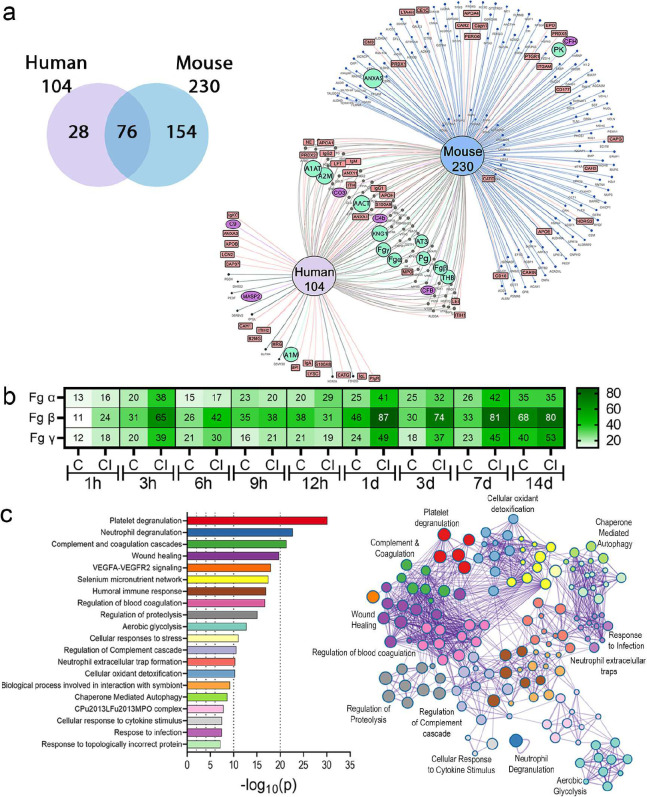
Proteomic analysis of the proteins deposited on urinary catheters retrieved from patients and mice. **(a)** Venn diagram comparing deposited proteins on urinary catheters retrieved from patients and mice catheterized with a dwell time of 1 day using E Venn online software^73^. Red rectangles represent inflammatory and immune proteins, green circles represent coagulation cascade proteins, purple ovals represent complement cascade proteins, while black dots represent other proteins. **(b)** Fg levels in acute and prolonged catheterized bladder in the absence or presence of *E. faecalis* OG1RF infection. **(c)** Metascape analysis showing the top significant pathways shared proteins found on mice and human catheters. Metascape network analysis and clusters of interactions of the top significant pathways were visualized with Cytoscape^74^.

Our previous immunofluorescence analyses on catheters showed increased Fg deposition during *E. faecalis* infection^16–18^, suggesting that infection may promote Fg recruitment. To test this, we performed a temporal quantitative proteomic analysis to compare Fg abundance on urinary catheters by analyzing the three Fg chains, α, β, γ, between catheterized (mock-infected) and catheterized and infected mice. Five catheters were harvested at the indicated timepoints, pooled, processed, and trypsin digested. Total protein analysis showed 230 proteins associated with mouse catheters (Fig. 2a,b, and Supplementary Table 1). Fg is among the most abundant deposited proteins on the catheter and its abundance increased in acute and prolonged infection starting 1 dpi (Fig. 2b). Higher Fg levels in infected bladders suggest that *E. faecalis* may have a mechanism to promote Fg accumulation.

### Coagulation and immune response pathway proteins are found on human and mouse urinary catheters.

**Extended Data Figure 4.**
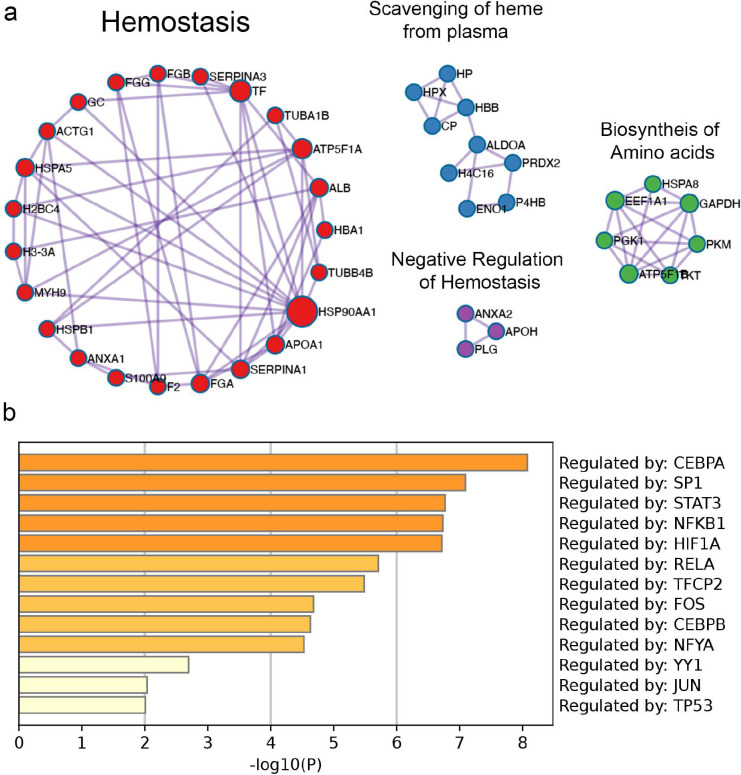
Protein-protein interactions and summary of transcriptional regulatory analysis. 76 shared mouse and human catheter proteins from Fig. 2 were analyzed on metascape.org^32^. **(a)** Molecular Complex Detection (MCODE) components identified from protein-protein interaction enrichment analysis. Generated networks were modified using Cytoscape network analysis and clusters of interactions were labelled by the top descriptive gene ontology for each protein-protein interaction^74^. **(b)** Summary of enrichment analysis of transcriptional regulatory relationships unraveled by sentence base text mining (TRRUST)^76^. Top Predicted transcriptional regulators were plotted based on −log10(P) values.

To understand if similar host proteins are deposited in human urinary catheter, we performed a proteomic analysis of 10 human urinary catheters with ~24 hrs dwell time, finding a total of 104 host deposited proteins, including several coagulation factors, including Fg, alpha-1 antitrypsin (A1AT), alpha-2-macroglobulin (A2M), plasminogen (Pg), antithrombin III (AT3), thrombin (THB), and kininogen (KNG) (Fig. 2a, Supplementary Table 1 and 2). Resembling mouse catheters, Fg is one of the most abundantly deposited protein. Comparative analyses showed 76 (73% of the identified human proteins) shared deposited proteins between mouse and human catheters (Fig. 2a). The 76 shared proteins were subjected to an unbiased and comprehensive pathway enrichment analyses using Metascape^32^, finding that the top significant pathways enriched are part of host inflammation, immune, hemostasis, and wound healing responses, including platelet degranulation, neutrophil degranulation, complement and coagulation cascades (Fig. 2a, Extended Data Fig 4a, and Supplementary Table 3). Furthermore, we found that transcriptional regulators involved in inflammation and immune responses, including CEBPA, STAT3, and NFKB1, were highly predicted in the catheterized bladder environment (Extended Data Fig 4b and Supplementary Table S4). Collectively, proteomics and ontology relationships indicate that coagulation and wound healing pathways are active in mouse and human catheterized bladders.

### Fibrinogen is critical for colonization of the catheterized bladder and catheter.

**Figure 3.**
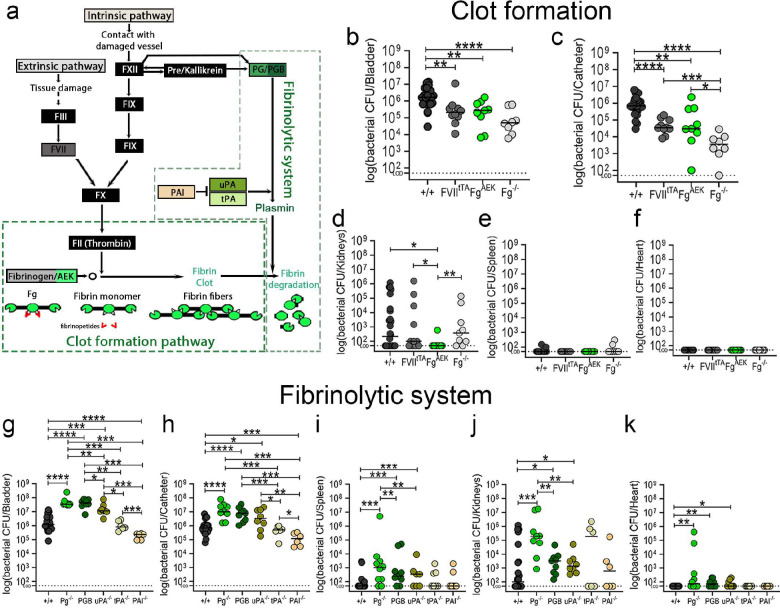
Impairment of the fibrinolysis enhances enterococcal colonization and systemic dissemination. **(a)** Coagulation cascade diagram (color boxes correlates with mouse strains used in this study). C57BL/6 wild type (WT) mice and transgenic coagulation mutants in C57BL/6-background looking at cloth formation pathway (**b-f**) or fibrinolytic system (**g-k**) were catheterized and infected with ~2×10^7^ CFU of *E. faecalis* OG1RF. After 24 hpi, bacterial burdens in bladder tissues (**a,g**), catheters (**b,h**), kidneys (**c,i**), spleen (**d,j**), and hearts (**f,k**). Values represent means ± SEM. The Mann-Whitney U test was used; *, P < 0.05 was considered statistically significant. **, P < 0.005; ***, P < 0.0005; ****, P<0.0001. The horizontal bar represents the median value. The horizontal broken line represents the limit of detection of viable bacteria. LOD; limit of detection. For CFU enumeration, infections were done in at least three independent experiments with n = 3–6 mice depending on the genotype, and data are shown as the log (bacterial CFU/organ or catheter). Animals that lost the catheter were not included in this work.

To investigate if Fg is critical for enterococcal CAUTI establishment, we compared bacterial colonization in C57BL/6 wild-type (WT) mice to several mutant mice (C57BL/6-background), including: i) Fg deficient mice (Fg^−/−^)^33^; ii) mice expressing a mutant form of Fg that cannot be converted to fibrin by thrombin, remaining a monomer (Fg^AEK^)^34^; and iii) FVIItTA/tTA (FVII_tTA_)^35^ mice, which are hypomorphic for coagulation factor VII (FVII)^36^, resulting in severe thrombin production downregulation, reducing fibrin formation (Fig. 3a). Mice were catheterized and infected; then at 1 dpi, bacterial burden was assessed in organs and catheters. Compared to WT mice, Fg^−/−^ mice exhibited significantly reduced enterococcal burdens in bladders (~1.5 logs) and catheters (~3.5 logs) (Fig. 3b,c). Similarly, Fg^AEK^ mice exhibited significant defects in bladder and catheter colonization (Fig. 3b,c). Fibrin reduction in the bladder via FVII^tTA^, resulted in significantly lower colonization in the bladders (~1 log) and catheters (~1.5 logs) (Fig. 3b,c). Soluble Fg presence (Fg^AEK^) significantly decreased kidney dissemination (Fig. 3d). Enterococcal dissemination to the spleen and hearts was not observed in any of the mouse lines (Fig. 3e,f). These results further confirm that Fg is critical for enterococcal CAUTI. Importantly, soluble Fg or low fibrin formation also results in lower colonization, indicating that fibrin accumulation is critical for persistent colonization.

### Fibrin accumulation enhances enterococcal bladder and catheter persistence during CAUTI.

Fibrinolysis is critical for dissolving fibrin net/clots and defective fibrinolysis results in fibrin accumulation. Based on this and that Pg was found on the human and mouse catheters (Fig. 2a), we investigated fibrinolysis in CAUTI, focusing in Pg and its key activators. We used C57BL/6-background mice deficient in different fibrinolysis factors (Fig. 3a). To increase fibrin accumulation, we used mouse lines deficient for: i) plasminogen (Pg^−/−^)^37^, ii) urokinase plasminogen activator (uPA^−/−^)^38^, and iii) tissue plasminogen activator (tPA^−/−^)^38^. To decrease fibrin levels, we used a deletion of plasminogen activator inhibitor (PAI^−/−^)^39^, which exhibits unregulated fibrin degradation (Fig. 3a).

We found that persistent fibrin accumulation led to significant increases of enterococcal bladder (~ 2 logs) and catheter (~1.5 logs) colonization in Pg^−/−^ mice when compared to WT mice (Fig. 3g,h). Importantly, fibrin reduction in the bladder via PAI deficiency, resulted in significantly lower bladder (~1 log) and catheter (~1.5 logs) colonization (Fig. 3g,h).

To further confirm that inactivation of Pg proteolytic activity correlates with enterococcal colonization enhancement, we used a transgenic mouse expressing Pg with a plasmin-inactivating active site mutation (PGB)^40^, finding that bacterial burden in the bladder and catheter was similar to Pg^−/−^ mice (Fig. 3g,h). This suggests that fibrinolysis inhibition correlates with exacerbating *E. faecalis* colonization.

Importantly, bladder and catheter bacterial colonization in uPA^−/−^ mice significantly increased similarly to Pg^−/−^ mice, whereas tPA^−/−^ mice exhibit similar bacterial burden to WT mice. Thus, uPA is the primary activator of Pg into plasmin in the catheterized bladder (Fig. 3g,h). Since coagulation dysregulation is not uncommon in human populations (**Supplementary Table 5**), these data suggest that coagulation deregulation could have a direct impact on enterococcal CAUTI.

### Host fibrinolytic deficiencies predispose the host to enterococcal systemic dissemination.

We further assessed the effect of Fg/fibrin modulation in enterococcal dissemination, finding that fibrinolytic defects (Pg^−/−^, PGB, and uPA^−/−^) significantly increased bacterial burden in kidneys, spleen, and hearts compared with WT mice colonization (Fig. 3i–k). Furthermore, tPA deficiency resulted in a bimodal kidney enterococcal colonization (Fig. 3i). Interestingly, kidneys with higher enterococcal colonization came from mice with higher bladder colonization, suggesting that a persistent bladder colonization may result in their dissemination (Fig. 3i). These demonstrate that fibrin accumulation predisposes the host to enterococcal systemic dissemination.

### *E. faecalis* proteases degrade plasmin and plasminogen but not thrombin.

We previously demonstrated that during CAUTI and growth in urine conditions, *E. faecalis* OG1RF induced the expression and activity of two enterococcal secreted proteases, a serine protease (SprE) and metalloproteinase gelatinase (GelE), which are critical for enterococcal CAUTI and systemic dissemination^29^. Given that *E. faecalis* in the catheterized bladder increases Fg/fibrin levels (Fig. 2b) and that fibrinolysis deficiencies enhance enterococcal colonization (Fig. 3), we examined if the enterococcal secreted proteases antagonizes the fibrinolytic system by assessing their ability to degrade human Pg (hPg) and plasmin (hPm) *in vitro.*

**Figure 4.**
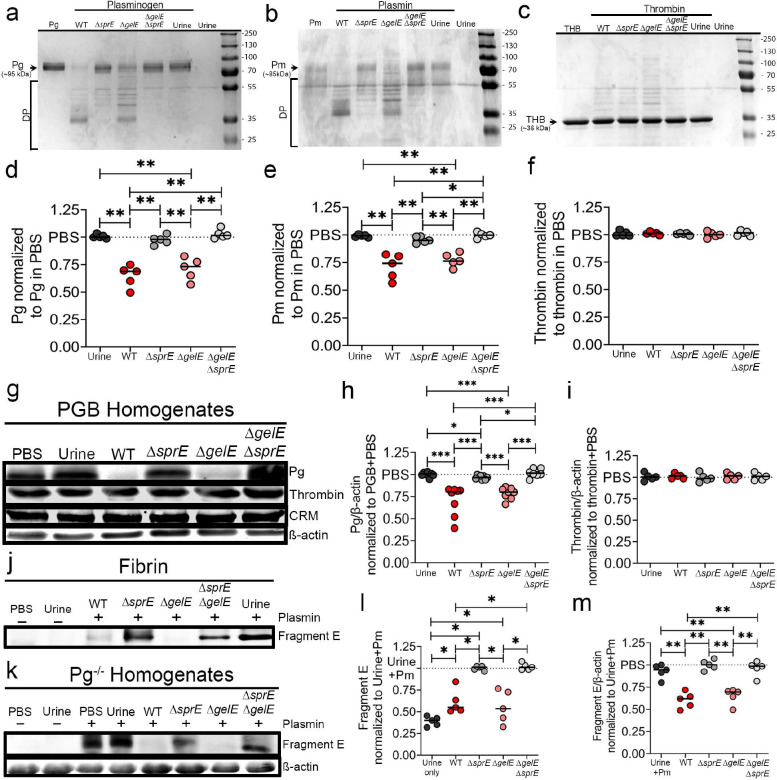
SprE, an *E. faecalis* secreted protease, selectively degrades plasminogen and plasmin, inactivating plasmin proteolytic activity against fibrin. **(a-c)** SDS-PAGE analysis of the proteolytic activity of *E. faecalis* WT and protease mutants’ cell-free supernatants against purified **(a)** plasminogen, **(b)** plasmin, or **(c)** thrombin and their corresponding degradation quantification by densitometry (**d-f**). **(g)** 24 hrs catheterized PGB bladder homogenates were incubated with *E. faecalis* WT and protease mutants’ cell-free supernatants and proteolytic activity against plasminogen and thrombin was monitored by Western blots. **(h-i)** Densitometry analysis of the Pg and thrombin degradation by bacterial supernatants performed in **(g)**. (**j-m**) SprE degradation of plasmin results in inhibition of fibrinolysis. To test this, supernatants *E. faecalis* grown in urine were filtered and concentrated, then incubated plasmin was for 4 hrs at 37°C; then each mixture was incubated with purified **(j)** fibrin or **(k)** Pg^−/−^ mouse bladder homogenates from 24 hrs catheterized non-infected mice. Degradation of fibrin was monitored by detection of Fragment E in incubation with fibrin or bladder homogenates by SDS-PAGE or western blot analysis, respectively **(j, k).** Fragment E quantification by densitometry obtained in **(l, m)**. CRM, cross reactive material. β-actin was used as loading and normalization control. Values represent means ± SEM. The Mann-Whitney U test was used; *, P < 0.05 was considered statistically significant. **, P < 0.005; ***, P < 0.0005. The horizontal bar represents the median value.

We compared the protease activity of WT, mutants defective for each protease (Δ*spr*E or Δ*gel*E), or both proteases (Δ*gel*EΔ*spr*E) against hPg and hPm under urine conditions (See Methods; **Supplementary Fig. 1**). Filtered WT supernatants degraded hPg (~95 kDa) and hPm (~85 kDa), producing a major degradation fragment ~35 kDa and 3 visible degradation products (DP) between 50–40 kDa (Fig. 4a,b). WT and Δ*gel*E supernatants produced similar hPg and hPm degradation pattern while the Δ*spr*E supernatant was not able to fully degrade hPg and hPm, showing three faint fragments between 50–40kDa and no major degradation product at ~35 kDa (Fig. 4a,b). The Δ*gel*EΔ*spr*E mutant completely lost the ability to degrade hPg and hPm into smaller peptides, similar to urine or PBS controls (Fig. 4a,b,d,e).

To understand whether these proteases are promiscuous and capable of targeting other factors in the coagulation cascade, specifically the fibrin formation pathway, we incubated the strains’ supernatants with thrombin, finding that thrombin was not degraded in any of the treatments (Fig. 4c,f).

To further validate these, we determined the proteolytic activity of supernatants from *E. faecalis* WT and various mutants against mouse Pg and thrombin in a complex catheterized environment. Cell-free bacterial supernatants were incubated with bladder homogenates (1:5) from PGB mice catheterized for 24 hrs (**Supplementary Fig. 1**). WT and Δ*gel*E supernatants significantly degraded Pg while no degradation was observed in the urine control, the Δ*spr*E single mutant and the double Δ*gel*E Δ*spr*E mutant supernatant (Fig. 4g–h). No cleavage of thrombin was observed with any of the treatments (Fig. 4g,i).

To determine whether *E. faecalis* proteases could functionally disrupt fibrinolysis by targeting plasmin, we incubated purified plasmin with WT and mutant strains’ supernatants, followed by a secondary incubation with thrombin-generated fibrin (Fig. 4j) or Pg^−/−^ homogenates (Fig. 4k) that were catheterized, non-infected, for 24hrs (**Supplementary Fig. 1**). Supernatants-containing SprE (WT and Δ*gel*E) degraded plasmin, resulting in lack of fragment E generation, demonstrating plasmin inhibition (Fig. 4j–m). These data suggests that *E. faecalis’* SprE protease activity in urine targets the fibrinolytic system by inactivating Pg and plasmin, which results in dysregulated fibrin accumulation.

### Fibrin accumulation rescues *E. faecalis* Δ*gel*EΔ*spr*E colonization deficiency.

**Figure 5.**
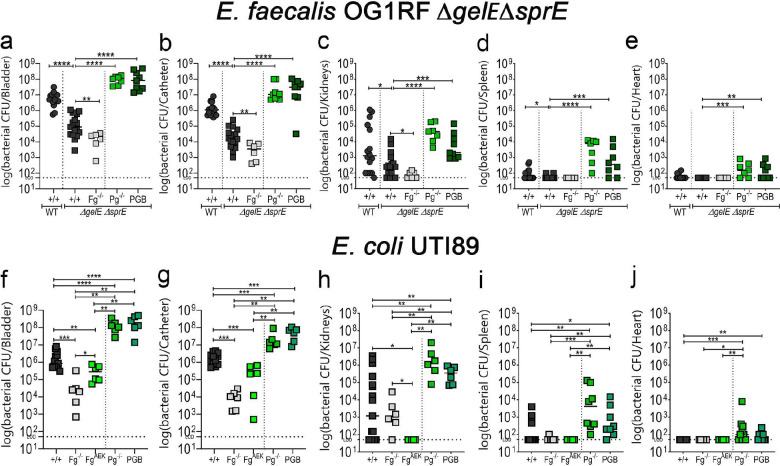
Host fibrinolytic deficiency rescued *E. faecalis* Δ*gel*EΔ*spr*E colonization deficiency, enhance colonization of uropathogen *E. coli* UTI89, promoting systemic dissemination. **(a-e)** C57BL/6 WT and coagulation deficient mice were infected with ~2×10^7^ CFU of *E. faecalis* OG1RFΔ*gelE*Δ*sprE* or (**f-j**) uropathogenic *E. coli* UTI89. After 24 hpi, bacterial burdens in bladder tissues (**a,f**), catheters (**b,g**), kidneys (**c,h**), spleen (**d,i**), and hearts (**e,j**). Values represent means ± SEM. The Mann-Whitney U test was used; *, P < 0.05 was considered statistically significant. **, P < 0.005; ***, P < 0.0005; ****, P<0.0001. The horizontal bar represents the median value. The horizontal broken line represents the limit of detection of viable bacteria. LOD; limit of detection. For CFU enumeration, infections were done at least in 3 independent experiments with n = 3–6 mice depending on the mouse genotype, and data are shown as the log(bacterial CFU/organ or catheter). Animals that lost the catheter were not included in this work.

If enterococcal protease actively modulates Fg and fibrin levels to enhance CAUTI colonization, then the colonization defect observed in the absence of protease activity should be reversed in mutant mice that accumulate fibrin. Expectedly, defective colonization of *E. faecalis* Δ*gel*EΔ*spr*E was further reduced in Fg^−/−^ mice. Predictively, defective colonization was rescued in mice that accumulate fibrin due to fibrinolytic deficiencies (Pg^−/−^ and PGB) (Fig. 5a,b), exhibiting higher than the WT strain colonization similar to fibrinolytic-deficient mice infected with WT strain (compare to Fig. 5a,b to Fig. 3). Importantly, fibrin accumulation in the catheterized bladder promoted *E. faecalis* Δ*gel*EΔ*spr*E systemic dissemination (Fig. 5c–e). This further demonstrates *E. faecalis*’ ability to modulate the coagulation cascade, creating an environment conducive to its colonization.

### Hosts with fibrinolytic deficiencies enhance uropathogen *E. coli* UTI89 CAUTI colonization and systemic dissemination.

Recently, we found that using a novel liquid-infused silicone catheter material to reduce Fg deposition on urinary catheters resulted in decreased bladder and catheter colonization and systemic dissemination by uropathogenic *E. coli* UTI89 in our mouse CAUTI model^19^. Here, we found that bladder and catheter colonization in Fg^−/−^ mice or coagulation-deficient mice (Fg^AEK^; soluble Fg) was significantly reduced (Fig. 5f,g). Conversely, not only was UTI89 bladder and catheter colonization significantly increased (~2 logs) in mice with fibrinolytic deficiencies (Pg^−/−^ and PGB) (Fig. 5f,g), but these mice also experienced significant systemic dissemination (Fig. 5h–j). These data provide evidence that uropathogenic *E. coli* colonization is modulated by Fg/fibrin in the catheterized bladder.

### Antifibrinolytic agent promotes a persistent CAUTI and systemic dissemination by diverse uropathogens.

**Figure 6.**
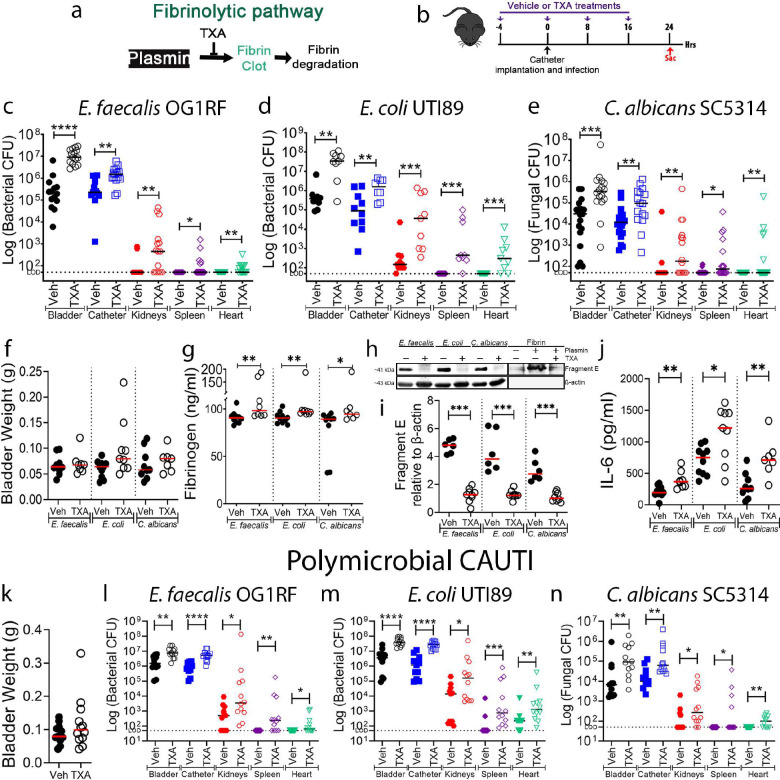
Pharmacological inhibition of plasmin proteolytic activity inhibits fibrin degradation and further enhances pathogen burden and dissemination of the top three CAUTI pathogens, *E. faecalis*, *E. coli*, and *C. albicans,* during mono- or polymicrobial infections. **(a)** Tranexamic acid (TXA) targets plasmin, thus inhibiting the fibrinolytic cascade. **(b)** Treatment timeline. **(c-e)** C57BL/6 WT mice were infected with ~2×10^7^ CFU of *E. faecalis* OG1RF **(c)**, ~2×10^7^ CFU of *E. coli* UTI89 **(d)**, or ~1×10^6^ CFU of *C. albicans* SC5314 **(e)** and dosed with either TXA (100 mg/mL i.p.) or vehicle (PBS). After 24 hpi, pathogen burdens were enumerated in bladder tissues, catheters, kidney pairs, spleens, and hearts. **(f)** Bladder edema was assessed after tissue harvest by weighing bladders. **(g-i)** Bladder homogenates were diluted (1:10) and analyzed for **(g)** fibrinogen, **(h-i)** fibrin degradation, and **(j)** IL-6. Fibrinogen and IL-6 levels were analyzed via ELISA. Fibrin degradation in the bladder homogenates was analyzed by measuring fragment E production via western blot and β-actin was used as loading and normalization control (**h**) and quantified by densitometry (**i**). **(k-n)** Polymicrobial CAUTI with *E. faecalis*, *E. coli,* and *C. albicans*. **(k)** Bladder edema of mice treated with TXA or vehicle during polymicrobial CAUTI. **(l-n)** C57BL/6 WT mice were infected with ~2×10^7^ CFU of *E. faecalis* OG1RF **(l)**, ~2×10^7^ CFU of *E. coli* UTI89 **(m)**, and ~1×10^6^ CFU of *C. albicans* SC5314 **(n)** and dosed with either TXA (100 mg/mL i.p.) or vehicle (PBS). Pathogen burden was assessed after 24 hpi in bladder tissues, catheters, kidney pairs, spleens, and hearts. Values represent means ± SEM. The Mann-Whitney U test was used; *, P < 0.05 was considered statistically significant. **, P < 0.005; ***, P < 0.0005; ****, P<0.0001. The horizontal bar represents the median value. The horizontal broken line represents the limit of detection of viable pathogen. LOD; limit of detection. For CFU enumeration, infections were done at least in 3 independent experiments with n = 3–6 mice each one, and data are shown as the log(microbial CFU/organ or catheter). Animals that lost the catheter were not included in this work.

Antifibrinolytic agents including tranexamic acid (TXA) are often used in patients with bleeding disorders or patients undergoing surgical procedures^41,42^ (**Table S3**). TXA is extensively used to treat patients with postpartum hemorrhages, traumatic injury, and surgical procedures that increase the risk of bleeding^42,43^. TXA is a synthetic anti-fibrinolytic amino acid that acts by competitively blocking the lysine binding sites on plasmin(ogen), inhibiting plasmin interaction with fibrin^43^ (Fig. 6a). As 86% of surgery patients require urinary catheterization^44^, we assessed whether TXA treatment during urinary catheterization increased fibrin accumulation in the bladder, enhancing colonization and systemic dissemination by the three most prevalent CAUTI uropathogens: *E. faecalis*, *E. coli*, and the fungal pathogen *C. albicans*. Mice were treated with TXA or vehicle control (PBS) intraperitoneally (Fig. 6b). Then, mice were catheterized and infected with the respective pathogen and sacrificed at 1 dpi. TXA-treated mice exhibited a significant increase of bladder and catheter colonization and promoted systemic dissemination of all pathogens when compared with PBS-treated mice (Fig. 6c–e). No significant differences in bladder weights between treatments were observed, suggesting that TXA did not exacerbate catheter-induced inflammation (Fig. 6f). To confirm fibrinolysis inhibition and fibrin accumulation, Fg/fibrin concentration and fragment E generation was compared and quantified between PBS- and TXA-treated bladders (Fig. 6g–i). We found that Fg/fibrin concentration increased and fragment E generation decreased in TXA-treated bladders (Fig. 6g–i). Since Fg induces IL-6 expression in immune and epithelial cells^45,46^, we measured IL-6 levels in the bladder. We found that IL-6 levels significantly increased in TXA-treated mice (Fig. 6j). This is consistent with increased Fg/fibrin levels in the TXA-treated bladders, suggesting a perpetual inflammatory positive-feedback loop in the catheterized host.

We further tested whether fibrin accumulation could enhance polymicrobial CAUTI. For this, TXA and PBS-treated mice were catheterized and infected with all three main uropathogens, *E. faecalis*, *E. coli*, and *C. albicans*. We found no difference in inflammation between PBS- and TXA-treated mice (Fig. 6k). However, blocking fibrinolysis with TXA significantly enhanced the burden of all pathogens in the bladders and catheters and increased systemic dissemination (Fig. 6l–n). In conclusion, these data show that pharmacological inhibition of fibrinolysis in the host enhances mono- and polymicrobial CAUTI and systemic dissemination by diverse uropathogens.

## Discussion

CAUTIs remain prevalent and costly, increasing patient morbidity and mortality^47,48^. CAUTI prevention and treatment faces major challenges, including the ability to predict: **i)** when catheterized patients develop infections; **ii)** which uropathogen will cause the infection; and **iii)** which patient population is more susceptible to septicemia. Previous attempts at treating CAUTIs include vaccine development, antibody therapy, and compounds that target specific pathogens^6,49^. However, multiple pathogens from different kingdoms can simultaneously cause CAUTIs and the risk of developing an infection increases with dwelling time. Efficiently predicting when patients will develop an infection, the causative organism, and which patient populations are more susceptible to systemic dissemination is imperative for successful CAUTIs prevention and treatment. Thus, a better understanding of CAUTI pathophysiology is critical to develop better clinical practices for improving patient outcomes.

Here, we found that the coagulation cascade plays an important role in CAUTI outcome. Importantly, we identified that host coagulopathies that result in fibrin clot accumulation promote higher pathogen colonization in the catheterized bladder by the most prevalent uropathogens in mono- or poly-microbial infections, enhancing persistence during CAUTI and promoting bloodstream infection and systemic dissemination (Fig. 3, 5, and 6). Fibrin is a major thrombus-formation end product^50^ and its concentration is tightly controlled by a series of cofactors, inhibitors, and receptors^15^. Once healing is resolved, the fibrinolytic pathway is triggered by activation of Pg into plasmin by uPA and tPA, promoting fibrin clot degradation and restoring tissue homeostasis (Fig. 3g)^14,15^. In the catheterized bladder, our results show that uPA, which was originally isolated from human urine, is the main Pg activator, but not tPA (Fig. 3). This could be related to their action mechanisms as tPA activation of Pg requires attachment to the fibrin on the clot surface to activate fibrin-bound Pg^51^. Since CAUTI pathogens form biofilms on fibrin, they may occlude tPA binding. In contrast, uPA is fibrin-independent and activates Pg in solution or when associated with its cellular receptor uPAR^52^, providing a cell-mediated Pg activation^53^. uPAR is present on many immune cells including macrophages^53^ that are recruited by urinary catheterization^20,27,28^; thus, it is possible that these immune cells are important for uPA-cell-mediated Pg activation. The uPA activation mechanism of Pg will require further experimentation.

Hypofibrinolysis and several thromboembolic diseases, including strokes, venous thromboembolism, rheumatoid arthritis, and renal diseases are accompanied by fibrin accumulation^54,55^. Furthermore, there are several congenital deficiencies that result in fibrin clot accumulation, including individuals with deficiencies of Pg, tPA, uPA, PAI-1, PAI-2, TAFI, and Annexin A2^50^ (**Table S3**). Consistent with our CAUTI data, fibrin accumulation from imbalance between activation of coagulation and inhibition of fibrinolysis, is detrimental for patients suffering from COVID-19^56^ and ventilator-associated pneumonia^57^. Moreover, fibrin accumulation can also result from antifibrinolytic treatments, which are commonly use in patients with bleeding disorders^55,58^ (**Table S3**), in patients with traumatic injuries, or undergoing surgical procedures that increase bleeding risk, including liver transplants, cardiac and cesarean surgeries ^41−43,59^. TXA is an active inhibitor of uPA and tPA, and also plasmin-fibrin binding^60,61^. Importantly, TXA elimination from the body is by urinary excretion, increasing its concentration in urine^59,60^. This information further validates our results showing that TXA is an efficient antifibrinolytic agent in the catheterized bladder, resulting in fibrin accumulation and enhancing CAUTI and higher systemic dissemination. Based on our data, these patient populations with coagulopathies or treatments that result in fibrin accumulation, if receiving a urinary catheter, may have a higher risk of developing a CAUTI and be more susceptible to secondary bacterial and fungal infection from a CAUTI.

IL-6 is the most highly elevated cytokine in the bladder, bloodstream, and liver and *E. faecalis* infection promotes its concentration (Fig. 1i). IL-6 is a potent regulator of inflammation, coagulation, and the immune response^62^ by regulating neutrophil infiltration, monocytes to macrophages differentiation, and Fg expression by hepatocytes^63,64^. Interestingly, Fg/fibrin also stimulates IL-6 expression by immune cells in the damaged tissue^53,65^; thus, resulting in a positive-feedback loop, dysregulated inflammation, and Fg/fibrin accumulation, promoting microbial colonization and perpetuating a futile inflammation cycle that benefits the pathogen. Importantly, IL-6/Fg dysregulation play a role in chronic inflammatory diseases and cancer^30,31,63^. IL-6 pathway activation was further supported by Metascape analysis showing that CEBPA and STAT3 are highly predicted transcriptional regulators of the catheterized bladder environment (Extended Data Fig. 4b). Interestingly, IL-6 is repressed during uUTI^28^, further demonstrating that uUTI and CAUTI have different pathophysiologies. Therefore, targeting IL-6/Fg signaling to modulate its effects on pathogenesis may provide a novel approach to improving CAUTI outcomes.

Here, we found that SprE actively degrades Pg and plasmin, suggesting that *E. faecalis* uses SprE to inactivate the fibrinolytic system. This strategy differs from other bacterial pathogens, including Group A Streptococcus (GAS), which activates Pg into plasmin via streptokinase, dissolving blood cloth to cause an invasive infection^66^. Therefore, identifying Pg/plasmin cleavage sites using purified SprE will be important to determine its action mechanism.

*E. faecalis* induction of fibrin accumulation creates ideal conditions for biofilm formation and persistence in the bladder, which is an open and dynamic system, where urine is constantly passing. Fibrin accumulation also contribute to the Fg/IL-6 feedback loop. This further explains how *E. faecalis* can thrive and exploit the catheterized bladder despite robust inflammatory responses and how fibrin accumulation is also conducive for CAUTI and systemic dissemination by other pathogens. Enterococci’s ability to modulate the bladder environment to benefit itself and other pathogens may further explain why enterococci are described as a pioneer species on urinary catheter. This supports several clinical studies that showed enterococci are early colonizers and persist during prolonged catheterization in monomicrobial or polymicrobial interactions^5,23,67^.

In uUTI*, E. coli* uses the type 1 pilus to bind mannosylated-receptors on the urothelial surface to invade and form intracellular bacterial communities (IBCs)^49^. During *E. coli* CAUTI, IBC formation is significantly reduced without affecting overall bladder colonization^68,69^. We found that Fg, which is highly glycosylated, is used by *E. coli* colonize the bladder and catheter during CAUTI^19^. Fg deposition onto the urothelium could block *E. coli-*cell interaction, affecting IBC formation. Consistent with this, we found that Fg/fibrin levels directly affected *E. coli* CAUTI (Fig. 5f,g), decreasing colonization when Fg was soluble or absent while increasing colonization when fibrin accumulated. Soluble Fg and other host glycosylated proteins, including uromodulin^69^, may act as decoy receptors to prevent binding to urinary tract surfaces, resulting in the pathogen’s expulsion by urine flow. In contrast, fibrin accumulation on surfaces can provide a platform for *E. coli* adherence to counteract its clearance by urine flow.

**Extended Data Figure 5.**
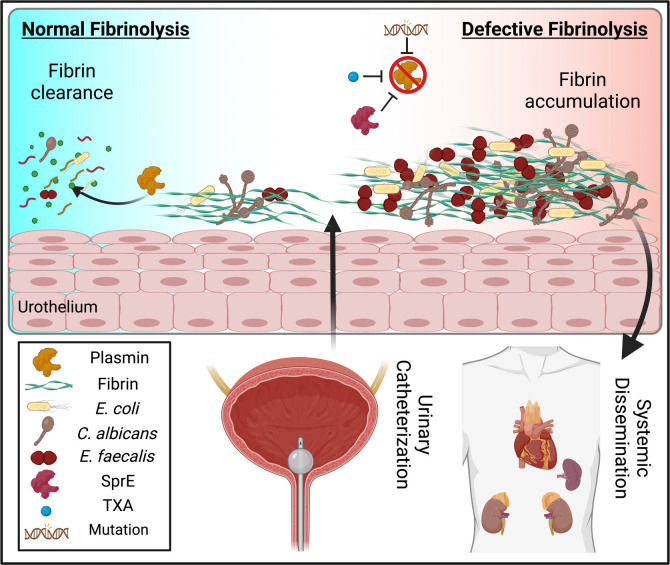
Fibrinolytic-deficiencies predispose hosts to severe CAUTI and septicemia by diverse uropathogens in mono- and poly-microbial infections. Defective fibrinolysis drives extravascular fibrin formation predisposing hosts to severe catheter associated urinary tract infections and systemic dissemination by prevalent CAUTI pathogens. *E. coli*, *E. faecalis*, and *C. albicans* each utilize fibrin for biofilm formation and virulence. Defective fibrinolysis results from congenital deficiencies, pharmacological inhibition through tranexamic acid, or by pathogen-secreted proteases such as *E. faecalis*’ serine protease SprE.

Given how common urinary catheterization is and the rise of multidrug resistant pathogens, the CAUTI frequency is expected to keep increasing. This study not only has identified mechanisms by which catheter-induced inflammation predispose patients to development of CAUTIs but also identified host populations that will be at higher risk of a CAUTI-associated septicemia caused by the most prevalent CAUTI pathogens (Extended Data Fig. 5). Our mouse CAUTI model has shown to faithfully recapitulate the pathophysiology of human CAUTI, suggesting that our findings have the potential to be translated for prevention and management of human CAUTI^6,17,18,21,49^. Leveraging these results to develop improved strategies for at-risk patient identification and to inform catheterization guidelines, will provide a higher patient quality of life and minimize the risk for complications.

### Resource availability

#### Lead Contact

Further information and requests for resources and reagents should be directed to and will be fulfilled by the lead contact, Ana Lidia Flores-Mireles (afloresm@nd.edu).

#### Materials availability

The study did not generate new unique material or reagents.

#### Data availability

- Proteomics raw data are available in supplementary tables 1 and 2. Raw data are available from lead investigator upon request.
- Any additional data required to reanalyze data presented in this article is available upon request.

### Ethics statement.

All animal care was consistent with the Guide for the Care and Use of Laboratory Animals from the National Research Council. The University of Notre Dame Institutional Animal Care and Use Committee approved all mouse infections and procedures as part of protocol #22-01-6971. For urine collections, all donors signed an informed consent form and protocol was approved by the Institutional Review Board of the University of Notre Dame under study #19-04-5273.

## Materials and Methods

### Experimental Model

#### Mouse Handling and Husbandry

Mice used in this study were ~6-week-old female wild-type C57BL/6 mice purchased from Jackson Laboratory and National Institute of Cancer Research or mutant mice bred in Harper Cancer Research Institute (Notre Dame, Indiana). The University of Notre Dame Institutional Animal Care and Use Committee approved all mouse infections and procedures as part of protocol number 22-01-6971. All animal care was consistent with the Guide for the Care and Use of Laboratory Animals from the National Research Council.

#### Mouse Infection Models

Mice were catheterized by placing a silicone catheter in the bladder through the urethra (transurethral) and infected as previously described^70^. Briefly, mice were anesthetized with isoflurane (inhalation) and implanted transurethrally with a 6-mm-long silicone tubing (Braintree Scientific SIL 025) which engulfed a 4mm-long PE tubing (BD Intramedic 427401). Mice were transurethrally infected with 50 μl of respective pathogen at an OD_600_ of 0.6 (~2 × 10^7^ CFU) or fungi at an OD_600_ of 5.0 (~1×10^6^ CFU) in PBS, or only catheterized. In triple infection experiments, the final CFUs/50 μl were ~2 × 10^7^ CFU for bacteria and ~1×10^6^ CFU for fungi. Immediately before sacrifice, blood was harvested via cheek poke and serum was extracted from whole blood (BD Microtainer #365967) for cytokine analysis as described below. To harvest catheters and organs, mice were sacrificed at their specified time-point by cervical dislocation after anesthesia inhalation; the bladder, kidneys, heart, spleen, liver, and if present, silicone catheter were aseptically harvested and bladders weighed. Except livers, all organs were homogenized (1min shake, 5 min rest, 1 min shake; MP Biomedical 116005500) and plated for CFU enumeration as described below. A subset of bladders used for histology analysis were weighed, fixed and processed as described below. Homogenized samples of bladder, liver and blood serum were sent for cytokine analysis as described below. Catheters were subjected to 15min sonication (Branson 2800) for CFU enumeration or sent for proteomic analysis as described below using nonimplanted catheters as controls. The University of Notre Dame Institutional Animal Care and Use Committee approved all mouse infections and procedures as part of protocol number 18-08-4792MD. All animal care was consistent with the Guide for the Care and Use of Laboratory Animals from the National Research Council^70^.

#### Microbial strains and growth conditions

Microbe strains used in this paper include *E. faecalis strains,* uropathogenic *E. coli*, and fungal *C. albicans* details listed in **Table S6**. Unless otherwise noted, all *E. faecalis* strains were grown overnight in 10 mL of brain heart infusion (BHI) media (Hardy Diagnostics C5143) at 37C in static conditions. *C. albicans* was grown in overnight in 10 mL of yeast-peptone-dextrose (YPD) media at 37°C in static conditions. *E. coli* strains were grown in LB (MP Biomedicals) shaking at 37°C for 4 hrs, diluted in LB (1:1000) then grown for 24 hrs in static conditions, then diluted once more (1:1000) and grown again for 24 hrs in static conditions.

#### Human urine collection

Human urine was collected and pooled from a minimum of two healthy female donors between the ages of 20 – 35 years to minimize donor variability. Donor health was indicated through lack of kidney disease, diabetes mellitus, or recent antibiotic treatment. Urine was filter-sterilized with a 0.22 μm filter (VWR 29186-212) and normalized to a pH of 6.0–6.5. All participants signed an informed consent form and protocols were approved by the local Internal Review Board at the University of Notre Dame under study #19-04-5273.

#### Collection of human urinary catheters

Patient catheters were collected with informed consent after the clinical decision to remove for standard of care was made as described^71^. This study was approved by the Washington University School of Medicine (WUSM) Internal Review Board (approval #201410058) and performed in accordance with WUSM’s ethical standards and the 1964 Helsinki declaration and its later amendments. Moreover, 10 catheters that had a dwell time of 24–26 hrs were further processed by the S.J.H. and M.G.C. laboratories for proteomics analysis.

#### H&E staining of mouse bladders and imaging

Mouse bladders were fixed in 10% neutralized formalin for 18hrs, embedded, sectioned, and stained as previously described^72^. Briefly, bladder sections were deparaffinized, rehydrated, and rinsed with water. Hematoxylin and Eosin (H&E) stain for light microscopy was done by the CORE facilities at the University of Notre Dame (ND CORE). All imaging was done using a Zeiss Axio Observer inverted light microscope. Zen Pro and ImageJ software were used to analyze the images.

#### Microbial Enumeration

Microbe load from bladder, kidney, spleen, hearts and silicone catheters from sacrificed animals was determined via serial dilution and enumeration. Organs were homogenized and silicone catheters were cut in small pieces before sonicated for CFU enumeration. Pathogens were plated in their corresponding media conditions (see *Microbial strains and growth conditions*).

#### Cytokine analysis

Bladder, liver, and blood serum samples from mice catheterized and infected with *E. faecalis* OG1RF or catheterized and mock infected with PBS for 1h, 3h, 6h, 9h, 12h, 1d, 3d, 7d, and 14d were frozen at −80 °C until time of assay. Before cytokine analysis, homogenates were thawed on ice and microcentrifuge at 11,000 × *g* for 10 min, and supernatants transferred to a new tube. Human urine was collected from patients catheterized for about 24h. Both mice and human samples were probed for IL-6, IL-1α, IL-1β, and TNFα levels using a Bio-Plex Multi-Plex Assay Kit from Bio-Rad Laboratories (Mouse 23-plex Assay Kit Cat# M60009RDPD and Human 27-plex assay kit M60009RDPD) following the manufacturer’s protocols.

#### Proteomic analysis of catheters

Donated catheters from patients catheterized for 24–26 hrs, or harvested catheters from mice that were catheterized and infected for 1hr, 3 hrs, 6 hrs, 1 day, 3 days, 7 days, and 14 days were boiled in SDS-sample buffer to release proteins. Protein samples were then processed by Proteomics and Mass Spectrometry Facility at the Danforth Plant Science Center (St. Louis, MO) as previously described^57^. Briefly, protein samples were trypsin-digested overnight at 37°C. The extracted peptides from each sample were dried and resuspended in 100 μL 1% ACN/1% FA. 5 μL were processed with an LTQ-Orbitrap Velos Pro on a Dionex RSLCnano HPLC using a 1h gradient. All MS/MS samples were analyzed using Mascot (Matrix Science, London, UK; version 2.5.1.0). Mascot parameters were set to search the cRAP_20110301 and NCBInr databases (selected for *Mus musculus* or *Homo sapiens* depending on catheter host) with a trypsin-digestion assumption. MS/MS peptide and protein identifications were validated with Scaffold (version Scaffold_4.4.5 Proteome Software Inc., Portland, OR). Protein identifications of mouse and human catheters that were for 1 dpi (mice) and 24–26 hrs dwell time (humans) were compared using an E Venn network^73^ online software (http://www.ehbio.com/test/venn/#/) to identify common proteins in the catheterized bladder environment between mice and humans. The 76-shared proteins were then submitted to metascape.org (**Table S3**) for gene ontology classification, gene ontology enrichment, protein-protein interaction identification, network analysis, and prediction of transcriptional regulatory relationships^32^. Generated networks were modified using Cytoscape software^74^.

#### Bacterial protease activity assay

Cultures of *E. faecalis* strains were centrifuged for 10 min at 3000x and washed three times with 1xPBS. Pellets were resuspended in 10ml of filter sterilized human urine at pH 6.5. The resuspended cultures were statically incubated for 18 hrs at 37°C. Following incubation, cultures were centrifuged and the supernatants were collected, filter-sterilized, and concentrated and size excluded using Macrosep Advance Centrifugal Devices (PALL MAP010C37). 40 μg/mL of purified protein (hPlasmin, hPlasminogen, or hThrombin) were added to supernatants and incubated statically for 4 hrs at 37°C. To determine proteolytic activity of *E. faecalis* strains in mice, urine-cultured supernatants were also added to PGB mouse bladder homogenates (1:5) that were catheterized only for 24 hrs. The mixture was incubated for 4 hrs at 37°C. Following incubation, 5x SDS sample buffer was diluted to 1x into the samples and boiled at 95°C for 2 minutes before separation on 12% acrylamide gels at 100V. Plasmin that was incubated with *E. faecalis* mutant strains’ supernatants was subsequently incubated with 25 μl of thrombin-generated fibrin (from 2 mg/mL of purified fibrinogen) or 25 μl of 24hpc Pg^−/−^ bladder homogenates for 4hrs at 37°C to determine functional disruption of fibrinolytic activity. Fibrin and homogenates incubated with supernatant-treated plasmin was then also processed for acrylamide gel protein separation. Normalization and western blots were done as described below.

#### Western blot

Western blotting was done as previously described^75^. Briefly, bladder homogenate samples were diluted 1:1 into 5X sample buffer then boiled at 95°C for 2 min. Following boiling, 10uL of sample in SDS was run on a 12% polyacrylamide gel for separation. Gels were then stained with Coomassie Blue for 60min followed by de-staining and imaging on an Odyssey infrared imaging system (LI-COR Biosciences, Lincoln, NE) to normalize protein concentrations. Following normalization, samples were again run on 12% acrylamide gels at 4°C and 50V. The samples were then transferred to polyvinylidene difluoride membrane using a semi-dry transfer (Millipore Sigma Cat# IPFL00005). Following transfer, membranes were blocked in 5% skim milk and probed by Western immunoblot with anti-β-actin (Abcam ab8229) as a loading control, and anti-plasminogen primary antibodies (Proteintech 17462-1-AP, respectively), anti-thrombin (ThermoFisher Scientific PA5-99213) or anti-Fg (Abcam ab34269) diluted in 3% skim milk in Tris-buffered saline-Tween (.2%). Membranes were then probed with secondary antibodies Donkey anti-goat (LI-COR Biosciences 925-68074) and Donkey anti-rabbit (LI-COR Biosciences 926-32213), diluted in dilution buffer (3% skim milk in .2% TBS-T, and .01% SDS) and visualized on an Odyssey CxL.

#### Measurement of Fg and IL-6 from bladder homogenates.

Bladder homogenates from respective mouse bladder groups were analyzed to determine abundances of Fg and IL-6 cytokine with respective ELISA kits following manufacture-provided protocols. Briefly, bladder homogenates were centrifuged and supernatants were diluted 1:10 in ELISA diluent. Dilution standard preparation was performed following manufacturer guidelines. Standards/samples were then placed on to pre-coated ELISA plates overnight at 4°C, removed, plates washed three times with wash buffer, incubated with detection antibodies for 1hr at room temperatures, washed three times, incubated with appropriate biotinylated-detecting enzyme for 30min at room temperature, washed 5 times, and incubated with appropriate chromogenic substrate for 15min at room temperature, and stopped with appropriate stop solution. Absorbances were measured at appropriate wavelengths with a plate reader (Molecular Devices Spectramax ABS plus).

#### Measurement of Fragment E

Fragment E was probed via SDS-PAGE and western blot with an anti-Fg primary antibody and anti-β-actin as a loading control. Fragment E probing was performed for the following experiments: **i)** *in vitro* bacterial protease activity of plasmin incubated with fibrin or with Pg−/− homogenates (Fig. 4j–m), **ii)** *in vitro* inhibition of plasmin fibrinolytic activity by TXA (**Fig. 7h**), and **iii)** homogenized bladders from both TXA-treated and vehicle controls (**Fig. 7h**). Fragment E band was visible at ~41kDa. Measurements of the median fluorescent intensity from each sample of both β-actin and fragment E lane were taken using image studio to determine densitometry. Measurements were graphed into Graphpad Prism 9 for statistical analysis

### Quantification and Statistical Analysis

#### Statistical Analysis

Data derived from this study was entered into Graphpad Prism 9 to generate statistical results and graphs. T-tests were used to determine significance between samples. When data was nonparametric, and the median better represented the distribution, comparisons were made with Mann-Whitney U Tests. Pearson’s correlation statistical analysis was used to measure association between variables.

## Extended Data

## Figures and Tables

**Key Resources Table:** 

Reagents or Resource	Source	Identifier
**Bacterial Strains**		
*Enterococcus faecalis* OG1RF	Murray et al., 1993	N/A
*Enterococcus faecalis Δspr*E	Thomas et al., 2009	N/A
*Enterococcus faecalis Δgel*E	Xu et al., 2017	N/A
*Enterococcus faecalis ΔsprE*/Δ*gel*E	Xu et al., 2017	N/A
*Escherichia coli* UTI89	Mulvey et al., 2001	N/A
*Candida albicans* SC5314	Odds, et al. 2004	N/A
**Chemicals, peptides, and recombinant proteins**		
Anti-beta Actin antibody	Abcam	ab8229
Anti-fibrinogen antibody	Abcam	ab34269
Donkey Anti-Goat IgG Polyclonal Antibody	LI-COR Biosciences	925-68074
Donkey Anti-Rabbit IgG Polyclonal Antibody	LI-COR Biosciences	926-32213
Dry Powdered Milk	Research Products International	M17200-100.0
Human Fibrinogen	Enzyme Research Laboratories	Fib 3
Human Fibrinogen ELISA Kit	Abcam	ab241383
Human Plasmin	Enzyme Research Laboratories	HPlasmin
Human Plasminogen	Enzyme Research Laboratories	HPg 2001
Human Thrombin	Sigma-Aldrich	T6884-250UN
Plasminogen polyclonal antibody	Proteintech	17462-1-AP
Phosphate Buffer Saline	Sigma-Aldrich	P3813-10PAK
Sodium Dodecyl Sulfate	Sigma-Aldrich	L3771-1KG
Thrombin polyclonal antibody	Fisher-Scientific	PA5-99213
Tranexamic Acid	VWR	TCA0236-100G
Tris-base	Fisher-Scientific	77-86-1
Tween-20	VWR	M147-1L
IL-6 Mouse Uncoated ELISA Kit	Invitrogen	88-7064-22
**Other Consumable**		
LB Medium	MP Biomedicals	3002-042
0.22 μm filter	VWR	29186-212
30G 1/2″ Needles	BD	305106
96-well plates, round bottom	Corning	3788
Agar	Hardy Diagnostics	C5003
BD Intramedic^TM^ Polyethylene Tubing	BD Intramedic	427401
BD Microtainer	BD	365967
Bio-Plex 23-Plex Assay Kit, Mouse	Bio-Rad	M500KCAF0Y
Bio-Plex 27-Plex Assay Kit, Human	Bio-Rad	M60009RDPD
Brain heart infusion (BHI) media	Hardy Diagnostics	C5143
Dextrose anhydrous	VWR	JT1919-1
Macrosep Advance Centrifugal Devices, MWCO 10 K	PALL	MAP010C37
Peptone	VWR	P20240-500.0
Polyvinylidene difluoride (PVD) membrane	Millipore Sigma	IPFL00005
RenaSil Silicone Rubber Tubing	Braintree Scientific	SIL 025
Yeast extract	MP Biomedicals	194027
**Organisms: mutant mice**		
C57BL/6 mice	NCI/Jax	000664
Fg^−/−^	Ploplis et al., 2000	N/A
Fg_AEK_	Prasad et al., 2015	N/A
Pg^−/−^	Ploplis et al., 2005	N/A
uPA^−/−^	Carmeliet et al., 1994	N/A
tPA^−/−^	Carmeliet et al., 1994	N/A
FVII_tTA_	Rosen et al., 2005	N/A
PAI^−/−^	Carmeliet et al., 2003 I/II	N/A
PGB	Iwaki et al., 2010	N/A
PG-_RKT_	Miles et al., 2017	N/A
FXII^−/−^	Pauer et al., 2007	N/A
PK^−/−^	Bird et al., 2012	N/A
KNG^−/−^	Yang et al., 2017	N/A
**Equipment**		
Zeiss Axio Observer	Zeiss	https://www.zeiss.com/microscopy/en/products/
FastPrep-24^TM^ 5G	MP Biomedical	116005500
Odyssey CLx Imager	LI-COR Biosciences	https://www.licor.com/bio/support/answer-portal/imaging-systems/odyssey-clx.html
Sonicator	Branson	2800
Spectramax ABS plus	Molecular Devices	ABSPLUS
**Software/Programs**		
Cytoscape	Shannon P. et al., 2003	https://cytoscape.org/
Evenn Venn network	Chen T. et al., 2021	http://www.ehbio.com/test/venn/#/
ImageJ	Schnieder et al.	https://imagej.nih.gov/ij
ImageStudio	LI-COR Biosciences	https://www.licor.com/bio/image-studio/
Metascape	Zhou Y. et al., 2019	https://metascape.org/
Prism 9	Graphpad	https://www.graphpad.com/
Zen Pro	Zeiss	https://www.zeiss.com/microscopy/en/products/software/
